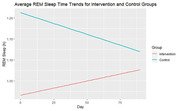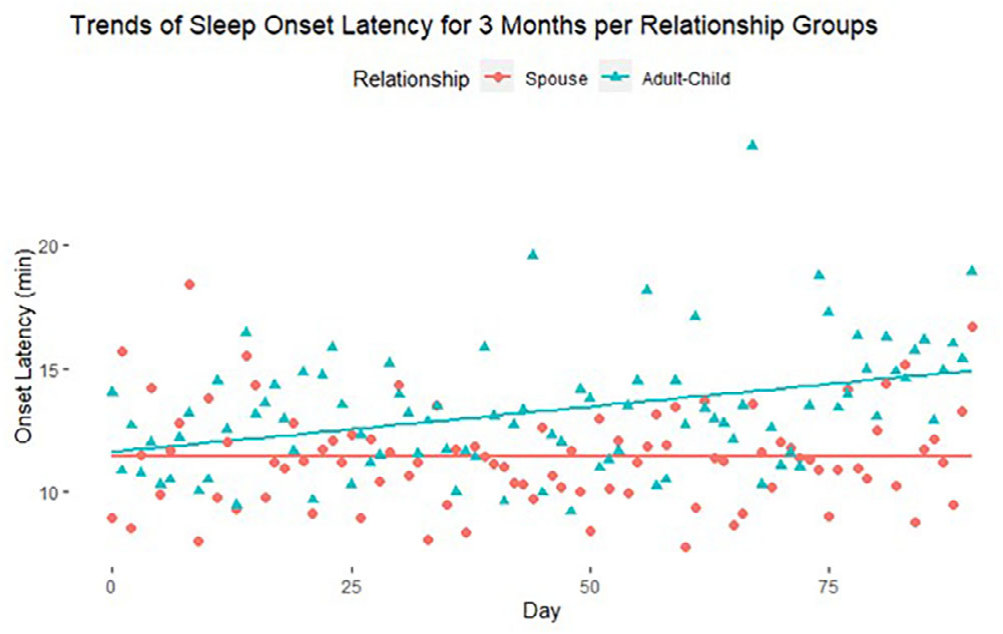# Wearable Internet‐of‐Thing Technology to Measure Sleep of Diverse Caregivers: Preliminary Results from a Randomized Controlled Trial

**DOI:** 10.1002/alz.092479

**Published:** 2025-01-09

**Authors:** Jung‐Ah Lee, Jiuchen Zhang, Amir Rahmani, Annie Qu

**Affiliations:** ^1^ University of California, Irvine, Irvine, CA USA

## Abstract

**Background:**

Family caregivers of persons with dementia (PWD) suffer from constant caregiving burden resulting in poor sleep quality. Understanding sleep parameters (e.g., total sleep, light, deep, REM sleep, awakening, latency) of the caregivers are important to improve sleep and self‐care behaviors. Wearable Internet‐Of‐Things (WIOT) technology (i.e., smartring‐smartphone‐cloud) would help monitor real‐time sleep quality of caregivers. WIOT is an objective tool to measure sleep parameters of caregivers with diverse ethnic background who might have limited English proficiency that could affect self‐report on survey sleep scales. The purpose of the study was to examine the effect of caregiver support intervention on sleep and to identify which characteristics of caregivers would get benefit from the intervention in relation to sleep.

**Methods:**

The study is a randomized controlled trial (RCT) including (1) a 3‐month culturally and language specific home‐based caregiver support intervention group (IG) and (2) attention control group (CG) without the intervention. All caregivers of PWD recruited from community outreach in California were asked to wear smartrings for 3 months to monitor their sleep in real‐time. Linear mixed models were performed using Python for data analyses including sleep and demographic variables.

**Results:**

103 caregivers participated in the RCT: mean age = 65 years (SD = 13), 78% females, 58% spouses, 53% with college education, race/ethnicity including 27% non‐Hispanic white, 16% Hispanic, 33% Korean, 24% Vietnamese. On daily average, REM sleep duration was significantly increased by 0.06 minute in IG and decreased 0.12 minute in CG (P<.001). In sub‐group analysis, average onset latency time were significantly improved in adult‐child caregivers compared to spousal caregivers (P = 0.016). The improvement of adult‐child caregivers’ sleep latency duration was in the reference range of 10‐20 minutes. Participants showed favorable interest in real‐time monitoring of their sleep through WIOT technology and better understanding of their sleep. Currently, the study participant recruitment is on‐going.

**Conclusion:**

The preliminary results showed that WIOT measures on sleep were feasible among ethnically diverse caregivers of PWD. The intervention effect on sleep was found among adult‐child caregivers. Intervention may focus more on support for spousal caregivers in using WIOT technology to better understand their sleep quality.